# Diagnostic Delay of Pulmonary Embolism in COVID-19 Patients

**DOI:** 10.3389/fmed.2021.637375

**Published:** 2021-04-30

**Authors:** Federica Melazzini, Margherita Reduzzi, Silvana Quaglini, Federica Fumoso, Marco Vincenzo Lenti, Antonio Di Sabatino

**Affiliations:** ^1^Department of Internal Medicine, San Matteo Hospital Foundation, University of Pavia, Pavia, Italy; ^2^Department of Electrical, Computer, and Biomedical Engineering, University of Pavia, Pavia, Italy

**Keywords:** pulmonary embolism, SARS-CoV-2, diagnostic delay, thrombosis, misdiagnosis

## Abstract

Pulmonary embolism (PE) is a frequent, life-threatening COVID-19 complication, whose diagnosis can be challenging because of its non-specific symptoms. There are no studies assessing the impact of diagnostic delay on COVID-19 related PE. The aim of our exploratory study was to assess the diagnostic delay of PE in COVID-19 patients, and to identify potential associations between patient- or physician-related variables and the delay. This is a single-center observational retrospective study that included 29 consecutive COVID-19 patients admitted to the San Matteo Hospital Foundation between February and May 2020, with a diagnosis of PE, and a control population of 23 non-COVID-19 patients admitted at our hospital during the same time lapse in 2019. We calculated the patient-related delay (i.e., the time between the onset of the symptoms and the first medical examination), and the physician-related delay (i.e., the time between the first medical examination and the diagnosis of PE). The overall diagnostic delay significantly correlated with the physician-related delay (*p* < 0.0001), with the tendency to a worse outcome in long physician-related diagnostic delay (*p* = 0.04). The delay was related to the presence of fever, respiratory symptoms and high levels of lactate dehydrogenase. It is important to rule out PE as soon as possible, in order to start the right therapy, to improve patient's outcome and to shorten the hospitalization.

## Introduction

Coronavirus disease 2019 (COVID-19) is caused by severe acute respiratory syndrome coronavirus (SARS-CoV-2), a novel coronavirus first detected in Wuhan City, Hubei Province of China in December 2019 (1). The Italian outbreak began in February 2020 and involved mainly Northern Italy, with Lombardy being on the front line (2). The COVID-19 pandemic progressively engulfed Europe and then most part of the world, with a massive impact on public health, politics and economics. As of October 20th, COVID-19 almost reached 40,300,000 cases with more than 1,115,000 deaths worldwide (3). Our academic tertiary referral hospital played a pivotal role in managing the emergency (4).

The clinical spectrum of this infection ranges from asymptomatic forms to multi-organ failure. According to a recent study that observed 5,700 COVID-19 patients hospitalized in the New York City area, the most common symptoms at admission were fever and tachypnea with dyspnea. Mortality rates ranged between 1.98 and 26.6% (in the 18-to-65 and older-than-65 age groups, respectively) and were significantly higher among patients who received mechanical ventilation (5).

SARS-CoV-2 infection can lead to severe complications such as acute respiratory distress syndrome (ARDS), acute renal failure, acute cardiac injury, and septic shock (6). Venous thromboembolism (VTE), that includes deep vein thrombosis (DVT) and pulmonary embolism (PE), is another potentially life-threatening complication reported in COVID-19 patients. An association between VTE and SARS-CoV-2 infection was first described by Zhai et al., who identified thrombotic events in 2.9% of a cohort of COVID-19 patients (7). In previous Asian series, thromboembolic events have been reported in roughly one fourth of COVID-19 patients admitted to the intensive care unit, and these findings correlated with a poor prognosis. Since then, dozens of papers on VTE incidence in COVID-19 patients have been published so far.

Generally, the diagnosis of PE can be challenging, mainly due to non-specific signs and symptoms, and diagnostic delay is common. Previous studies described an average time between symptom onset and PE diagnosis that varied from 4.8 to almost 9.0 days (8–10).

Due to the wide and partially overlapped clinical spectrum of both COVID-19 and PE, the differential diagnosis of these conditions can be demanding. Moreover, it is possible that the novelty of the situation and the lack of knowledge about this new infection led the clinicians to overlook the diagnosis of severe comorbidities, such as PE.

On this basis, we hypothesized that PE in COVID-19 patients could be misdiagnosed or underdiagnosed, determining a diagnostic delay that could affect the prognosis. The presenting signs and symptoms can be tricky and subtle, thus contributing to the delay. The identification of specific features of the PE related to COVID-19 could help the clinician to discriminate which patients should be promptly evaluated for PE.

The primary aim of our study was to assess the diagnostic delay by analyzing data from the clinical records of hospitalized COVID-19 patients with PE, in comparison to hospitalized non-COVID-19 patients with PE. The secondary aim was to identify a potential association between patient- or physician-related variables and the delay.

## Materials and Methods

### Patient Population and Study Design

This was an exploratory, single-center observational retrospective study conducted in an academic, tertiary hospital in Pavia, Italy (San Matteo Hospital Foundation).

The study included all consecutive COVID-19 patients admitted to the San Matteo Hospital Foundation between February 2020 and May 2020, in which a diagnosis of PE was confirmed with angiographic computed tomography (CT). Patients below 18-year-old at the time of diagnosis were excluded. A control population of non-COVID-19 patients with a confirmed diagnosis of PE admitted at our hospital during the same time lapse in the previous year (February 2019–May 2019) entered into the study.

In each case, requested data were obtained from the local electronic records of the San Matteo Hospital Foundation, anonymized and then entered into a database. We reviewed the clinical history of each patient, looking for all the possible presenting signs, symptoms, and clues that were related to PE and COVID-19 onset, according to the present literature and expert opinion. To note, regarding COVID-19 patients, all data regarding the onset of symptoms were accurately collected at the time of, and during, hospitalization by the treating physicians. Asymptomatic patients with an incidental finding of PE (e.g., angiographic CT performed for oncological follow-up) were excluded. Furthermore, we reported relevant sociodemographic features, comorbidities, risk factors for thrombosis and outcomes (i.e., dead or discharged). The number of physicians involved in the diagnosis of PE, as well as the possible misdiagnoses, were also indicated. Among the blood tests, platelet count (PC), lactate dehydrogenase (LDH), and D-dimer at admission were recorded. If not performed at admission, laboratory tests were taken into account only if performed within the first 48 h.

For the purpose of the study, we considered two types of delay. The patient-related delay, defined as the time between the onset of the symptoms and the first medical examination, and the physician-related delay, defined as the time between the first medical examination and the final diagnosis of PE. The overall diagnostic delay was obtained by summing both patient-related and physician-related delay and expressed in days. The day of the diagnosis of PE was considered as the date of the pulmonary angiographic CT.

The study was performed as a clinical audit using routinely collected clinical data and as such is exempt from the need to require written informed consent. The study was approved by the local ethics committee (San Matteo Hospital Foundation; Protocol Number 2020-0072882).

### Statistical Analysis

The RStudio (11) statistical package was used for all the descriptive and inferential statistics. Median and range were used instead of mean and standard deviation due to skewed data distributions. Non-parametric Wilcoxon-test was used to check the difference between continuous variables. Kaplan-Meier estimate was used to plot cumulative diagnosis probability in patients who died or were alive at discharge. Log-rank-test was used to assess the difference between survival curves. Pearson's correlation coefficient has been used to measure linear correlation between pairs of variables. Univariable and multivariable linear regression analyzes were used to find predictors of the diagnostic delay. For the univariate, the most frequent and important variables used in the current literature were analyzed. For the multivariate analysis, none authomatic procedure was used. Variables that were significant at univariate analysis were considered first, then additional variables have been tested since also those that are not significant at univariate analysis could show significance once combined with other ones. Moreover, we limit the set of the tested models to three variables, given our limited sample size, using the rule of thumb of around 10 cases for variable. Logarithmic transformation of the observed delay times was done in order to improve the times distribution normality. Given the relatively small sample size, we decided to test multivariate models with no more than three variables, to avoid overfitting.

## Results

The PE-associated COVID-19 population comprised 29 patients, with a median age at diagnosis of 62 years (range 29–82, M:F ratio = 3.8:1). Other sociodemographic features included in the study are shown in Table 1, which also show the same variables for the control population (non-COVID-19 patients).

**Table 1:** Sociodemographic characteristics of the 29 COVID-19 patients with PE and 23 non-COVID-19 patients with PE.

	**COVID-19 (2020)**	**Non-COVID-19 (2019)**
	***n* (%)**	***n* (%)**
**Age**		
≥65	10 (34.5)	15 (65.2)
** <65**	19 (65.5)	8 (34.8)
**Sex**		
**Female**	6 (20.7)	12 (52.2)
**Male**	23 (79.3)	11 (47.8)
**Smoking status**		
**Never smoked**	13 (44.8)	11 (47.8)
**Current smoker**	5 (17.2)	7 (30.4)
**Former smoker**	11 (38)	5 (21.8)
**Obesity (BMI** **≥** **30)**		
**Yes**	2 (6.9)	3 (13)
**No**	27 (93.1)	20 (87)
**Years of education**		
≤5	0	1 (4.3)
>5, ≤8	3 (10.3)	3 (13)
>8, ≤13	11 (38)	10 (43.5)
**>13**	15 (51.7)	9 (39.2)
**Marital status**		
**Single or divorced**	8 (27.6)	4 (17.4)
**Married**	17 (58.6)	15 (65.2)
**Widowed**	3 (10.3)	4 (17.4)
**Cohabiting/partner**	1 (3.5)	0
**Exemption from healthcare taxes**		
**No**	8 (27.6)	4 (17.4)
**Yes**	21 (72.4)	19 (82.6)
**Income**		
** <1,000** €	14 (48.3)	13 (56.5)
≥1,000 €	15 (51.7)	10 (43.5)

*PE, pulmonary embolism*.

*The included variables are in bold*.

In the COVID-19 population the median overall diagnostic delay was 19 days (range 1–47), the median patient-related and physician-related delay were, respectively, 3 days (range 0–10) and 14 days (range 0–46).

All patients showed COVID-19 related symptoms, being the most frequent clinical pictures fever with dyspnea (13 patients, 44.8%), fever with dyspnea and cough (five patients, 17.2%), fever with dyspnea and gastrointestinal symptoms (four patients, 13.8%).

Signs, symptoms and clues potentially related to PE are shown in Table 2.

**Table 2:** Symptoms, alterations, or clues that have prompted further work-up to confirm pulmonary embolism in COVID-19 patients.

	***N* (%)**
**Respiratory symptoms**	
Dyspnea not requiring oxygen therapy	5 (17.2)
Dyspnea requiring oxygen therapy	9 (31)
Dyspnea requiring mechanical invasive ventilation	15 (51.7)
Cough	8 (27.6)
Hemoptysis	2 (6.9)
**Heart symptoms**	
Thorax pain	4 (13.8)
Palpitations	0
Syncope	0
**Fever**	26 (89.7)
**Hematological alterations**	
Increase in platelets number (>400 × 10^9^/L)	1 (3.5)
Decrease in platelets number (<150 × 10^9^/L)	5 (17.2)
Increase in LDH levels (>220 mU/mL)	26 (89.7)
Increase in D-dimer levels (>500 mcg/L)	11 (37.9)
Increase in D-dimer levels (>5,000 mcg/L)	11 (37.9)
**Deep vein thrombosis**	17 (58.6)

*LDH, lactate dehydrogenase*.

Dyspnea was always present, with various degrees of severity. Seventeen out of 29 patients (58.6%) had DVT at the time of SARS-CoV-2 infection diagnosis. D-dimer levels were altered in 22 patients (75.8%). Other blood tests revealed thrombocytopenia in five patients (17.2%), thrombocytosis in one patient (3.5%). LDH was abnormal in 26 patients (89.6%).

Almost all the patients were first assessed by an emergency physician (27 patients, 93.1%). In 26 cases (89.6%) at least another physician was consulted in the diagnostic process. In the majority of cases, the reason for the achievement of the definite diagnosis was a persistent respiratory failure (16 patients, 55.2%). Other clues that led to the diagnosis were: increased levels of D-dimer (eight patients, 27.6%), compression ultrasonography screening (1 patient, 3.4%), incidental finding (four patients, 13.8%).

When present (18 patients, 62.1%), the misdiagnosis was mostly a worsening of the COVID-19 related pneumonia (13 patients, 44.8%). Further misdiagnoses are described in Table 3.

**Table 3:** Misdiagnosis that led to diagnostic delay.

	***N* (%)**
**Respiratory diseases**	
Worsening in COVID-19 pneumonia	13 (44.8)
Bacterial superinfection	2 (6.9)
COPD exacerbation	1 (3.5)
**Heart diseases**	
Acute pulmonary edema	2 (6.9)

*COPD, chronic obstructive pulmonary disease*.

Notably, only a few patients (10 patients, 34.4%) had previous thrombotic risk factors. The median number of comorbidities was 2.5 (range 0–10).

The outcome was positive (patient alive upon discharge) in 19 patients (65.5%) and negative (death) in 10 patients (34.5%).

**Figure 1:**
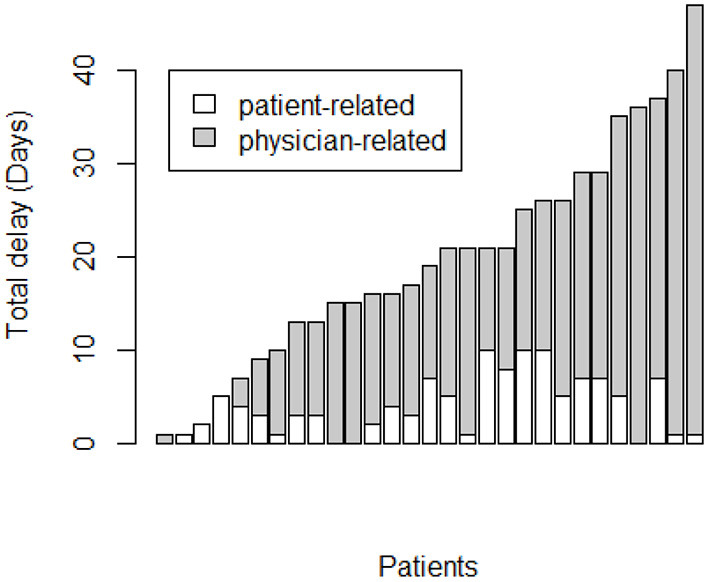
Correlation between pulmonary embolism overall diagnostic delay (days) and physician-related delay.

The overall diagnostic delay depends mainly on the physician-related delay that is significantly higher than patient-related delay (14 days, range 0–46 vs. 3 days, range 0–10, *p* < 0.0001) (Figure 1).

**Figure 2:**
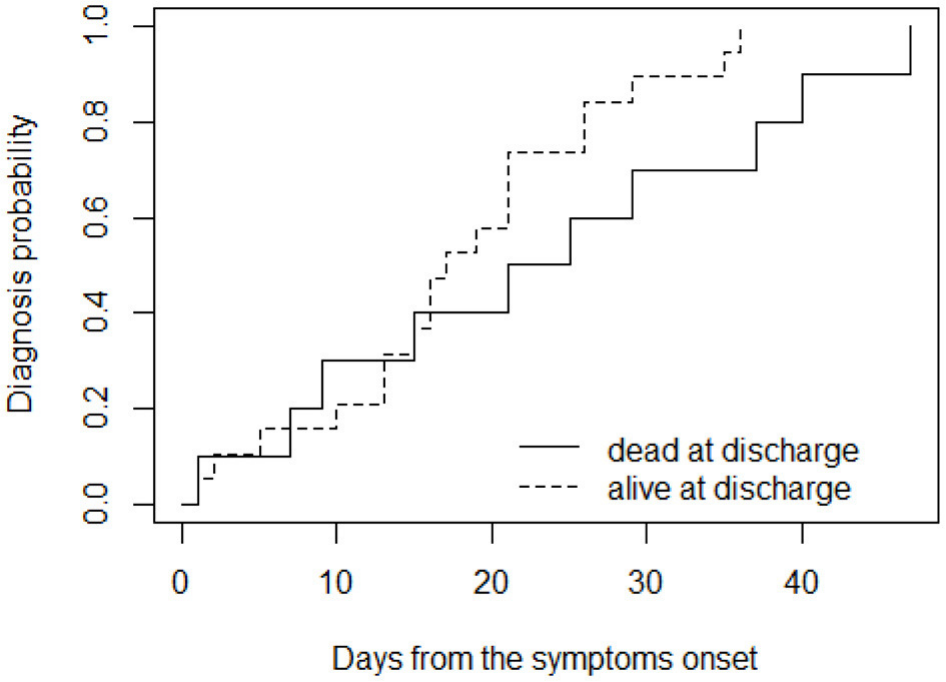
Cumulative probability of receiving diagnosis over time, for patients grouped according to their outcome at (dead or alive) at discharge.

Moreover, considering long diagnostic delay (≥10 days), the delay was significantly higher in patient who died (*p* = 0.02; Figure 2).

Univariable and multivariable analysis were performed to identify the factors that affected the diagnostic delay the most (Table 4).

**Table 4:** Univariable and multivariable analysis for the most relevant characteristics considered for overall, physician-dependent and patient-dependent diagnostic delay in COVID-19 patients affected by pulmonary embolism.

**Diagnostic delay**		**Univariable analysis**		**Multivariable analysis**	
**Overall**	**Median (dy; 25th−75th)**	**Difference in log (95%CI)**	***P*-value**	**Difference in log (95%CI)**	***P*-value**
**Sex**					
Female	20 (8.5–24.75)	0	0.431		
Male	17 (13–27.5)	0.34 (−0.50 to 1.19)			
**Fever**					
Yes	21 (15–29)	1.72 (0.95 to 2.45)	0.00016	1.56 (0.67 to 2.47)	0.0023
No	3.5 (1–7)	0			
**Dyspnoea**					
Mild	14 (6–20)	0			
Severe	25 (17.5–35.5)	0.94 (0.34 to 1.53)	0.00473	0.58 (0.06 to 1.10)	0.040
**D-dimer elevation**					
Yes	13 (8–15.25)	−0.7506 (−1.35 to −0.15)	0.0206		
No	21 (16–29)	0			
**LDH**					
Normal	29 (25–33)	0			
High	16.5 (13–26)	−0.6 (−1.85 to 0.65)	0.356	−0.59 (−1.12 to −0.05)	0.041
**Physician-dependent**					
**Sex**					
Female	12.5 (3–15.25)	0	0.20145		
Male	15 (10–21.5)	0.6316 (−0.31 to 1.58)			
**Fever**					
Yes	15 (12–22)	1.9517 (1.07 to 2.83)	0.00017	1.56 (0.54 to 2.58)	0.006
No	0 (0–2.5)	0			
**Dyspnoea**					
Mild	11.5 (1.5–14.75)	0			
Severe	16 (12–30)	0.9995 (0.30 to 1.69)	0.00889	0.53 (0.11 to 0.94)	0.02
**LDH**					
Normal	15 (15.75–25.25)	0			
High	20.5 (10–20.75)	−0.000570 (−0.00296 to 0.00096)	0.6	−0.001793 (−0.00296 to 0.00096)	0.03
**DVT**					
Yes	13 (10.5–15.5)	0.0733 (−0.98 to 1.12)	0.892		
No	13 (9–20)	0			
**Risk factor VTE**					
Yes	12.5 (2.5–15)	−0.6520 (−1.2 to −0.05)	0.033		
No	15.5 (12–28)	0			
**Physician specialization**					
ED	0	−2.5185 (−3.66 to −1.37)	0.000238		
Internal medicine	14 (7–17)	−0.3699 (−1.51 to 0.77)	0.532308		
Pulmonology	14 (9.75–17.25)	0.1225 (−0.88 to 1.13)	0.813249		
Infectious diseases	13.5 (11.5–15.5)	0			
ICU	18.5 (12–30)	0.4267 (−0.52 to 1.37)	0.386113		
**Previous misdiagnosis**					
No	13 (0–14)	(−39.7 to 52.7)	0.019273		
Interstitial pneumoniae	11.5 (10.5–15.5)	(−26.54 to 40.87)	0.001180		
Worsening i. p.	22 (13.5–33)	(−24.71 to 41.06)	0.000296		
**Patient-dependent**					
**Sex**					
Female	7 (2–7.5)	0	0.336		
Male	3 (1–5)	−0.3315 (−0.69 to 0.62)			
**Years of education**		(−0.62 to 0.22)	0.361		
≤5					
≤8	8 (6–9)	0			
≤13	2 (1–4.5)	−0.8661 (−1.78 to 0.05)	0.07		
>13	3 (1–6)	−0.6970 (−1.59 to 0.19)	0.14		
**Dyspnoea**					
Mild	2 (1–4.5)	0			
Severe	5 (2–7)	0.28 (−0.26 to 0.81)	0.322		
**D-dimer**					
D-Dimer <5,000 mcg/L	3 (1–5)	0			
D-Dimer ≥5,000 mcg/L	5 (2.75–7)	0.55 (−0.04 to 1.13)	0.07		

*DVT, deep vein thrombosis; ED, emergency department; ICU, intensive care unit; LDH, lactate dehydrogenase; VTE, venous thromboembolism; i.p., interstitial pneumonia*.

A lower level of education was statistically associated with a longer patient-related delay, while the presence of fever with a longer physician-related delay. When the definitive diagnosis was based upon an increase in D-dimer levels, the physician delay remarkably decreased.

The variables that only showed a trend toward a statistically significant prolonged delay, though without reaching it, were female sex and single/divorced/widowed status for the patient-related delay, and the number of specialists involved in the diagnosis for the physician-related delay.

Age, monthly income, exemption from medical expenses, levels of LDH or PC, presenting symptoms (either COVID-19 symptoms or PE symptoms, including DVT), thrombotic risk factors and comorbidities did not influence the patient-related delay. Furthermore, no association was found between physician-related delay and level of instruction, gender, marital status, specialization of first medical consultant, comorbidities, thrombotic risk factors, misdiagnosis, blood tests.

According to the multivariable analysis, the key factors for the delay were the presence of fever, respiratory symptoms and high levels of LDH. When considered together, these factors remained extremely relevant and determined a prolonged delay. The correlation was significant both for the physician-related delay and for the overall diagnostic delay.

The control population included 23 patients with PE but without COVID-19. Sociodemographic characteristics are described in Table 1. There were no significant differences between the 2 groups, except from higher prevalence of male patients in the COVID-19 group (*p* = 0.03) and a slight older age in non-COVID-19 patients (*p* = 0.05). Among clinical and laboratory findings, three patients (13.0%) referred palpitations and 14 patients (60.8%) presented altered LDH levels. In 20 patients (86.9%) the specialist that first assessed the patient was an emergency doctor, who was also the physician that most frequently established the correct diagnosis (15 patients, 65.2%). The feature that led to the diagnosis in most of the patients (13 patients, 56.5%) was the persistence of respiratory failure.

The median overall diagnostic delay in this population was seven days (range 0–30). Of note, the median physician-related delay was only 4 days (range 0–30), while the median patient-related delay was 0 days (range 0–26).

No correlation was found between the diagnostic delay and level of education, marital status, gender, age at diagnosis, monthly income, exemption from medical expenses, LDH, PC and D-dimer, presenting symptoms including DVT, comorbidities, specialization of specialists involved in the diagnosis. Instead, a higher number of specialists corresponded to a longer physician-related delay.

Differently from the COVID-19 cohort, where the overall diagnostic delay was only physician-related, in the non-COVID-19 cohort, it was statistically correlated with both the physician-related delay (*p* = 0.011) and, even more, with the patient-related delay (*p* < 0.0001).

## Discussion

COVID-19 is a recently described disease, the possible clinical scenarios of which are still under investigation. Pulmonary embolism is a widespread, life-threatening condition that can complicate COVID-19. Both pathologies may exhibit unspecific and partly overlapping signs and symptoms, with a consequent diagnostic delay. Our study sought to investigate this diagnostic delay, identifying any characteristics that may early identify patients who need to be evaluated for this important comorbidity.

The median overall diagnostic delay was 19 days for the PE-COVID-19 cohort, while in non-COVID-19 patients was 7 days.

The presence of a significant diagnostic delay in patients with PE has previously been demonstrated in other papers (8–10). However, our study, which specifically analyzed patients with PE and COVID-19, showed that the diagnostic delay is even greater in the case of SARS-CoV-2 infection. The median overall delay in PE-COVID-19 patients was 19 days, twice as long as the delay reported by Bulbul et al. and Wallen et al. (8.4 and 9 days, respectively) (8, 10) and the quadruple compared to the delay described by Elliott et al. (4.8 days) (9).

Analyzing the two different components of the PE-COVID-19 delay, physician and patient-related, respectively, it emerged how the delay was almost exclusively attributable to the physicians. The main feature that led physicians to diagnostic delay was the presence of fever. This is quite understandable since fever is a non-specific symptom that is frequent in various pathological conditions including infections, particularly COVID-19. In addition, fever is a rare clinical presentation of PE (12). Even if it is difficult to prove it, we can also speculate on the fact that the state of emergency and the novelty of this condition have negatively influenced the work of physicians. According to current knowledge, it is certainly a mistake not to consider PE as a possible diagnosis just for the presence of fever. This retrospective study analyzed COVID-19 patient's management in the first period of the pandemic, when the correlation with thrombotic phenomena was still based on few studies.

The physician-related delay was instead greatly decreased when the clue that led to the diagnosis was an increase in the D-dimer values. This is also understandable, given that this data is considered to be more specifically indicative of PE, or generally VTE (13–15). Therefore, it seems reasonable to support the D-dimer screening in COVID-19 patients, both at admission and during hospitalization.

The patient-related delay was significantly increased in patients with a low level of education. Probably the lack of instruction made it possible for the patients not to identify some clinical characteristics as dangerous and indicative of a pathological condition.

Even if it was not statistically significant, the presence of DVT conducted the patient to quickly look for a medical examination. Definitely, DVT often has visible, typical and disabling signs and symptoms.

During the COVID-19 epidemic, people were found to be afraid of going to hospital, even in the presence of alarm symptoms, due to a potential infectious risk. This is supported by the dramatic increase of out-of-hospital cardiac arrests that were noticed in Lombardy during the highest peak of infections (16). This may have caused an important patient-related diagnostic delay for various diseases, including non-COVID-19 related diseases.

Fortunately, a longer diagnostic delay was not statistically related to a worse outcome, although there was a tendency, which was observed for longer delays. Even if one study reported a higher in-hospital mortality rate in patients with a diagnostic delay >3 days (17), most of the other studies are in line with our results (18–21). Also, all hospitalized COVID-19 patients were given thromboprophylaxis with heparin, and this could have improved the final outcome.

Dyspnea as the main clinical symptom has been previously associated with a shorter time to diagnosis (19). In other cases, this association was not found (10), or even the presence of dyspnea led to a longer diagnostic delay (22). In our series, dyspnea was reported in all the patients; therefore, it is difficult to understand its role in the diagnostic delay. Thus, its meaning remains controversial in the differential diagnostic process.

Notably, the presence of pre-existent risk factors for VTE did not reduce the delay so much, while this association was reported in many other studies (17, 18, 22, 23).

In non-COVID-19 patients the median diagnostic delay was only 7 days, even lower than the median diagnostic delay in the general patient population (8–10). This can reasonably exclude a systematic medical error in the diagnosis of PE in our hospital. Although the delay in this cohort was statistically related to a delay of both the physician and the patient, the latter was the component that affected the delay the most.

The statistical analysis revealed that the overall delay in COVID-19 patients was significatively longer than in non-COVID-19 patients. Moreover, the correlation between the overall delay and the physician-related delay was more pronounced for the COVID-19 patients.

Some differences in the 2 groups of the study must be mentioned. As expected, male were more represented in COVID-19 cohort. The age of the non-COVID-19 patients tended to be higher than that of COVID-19 patients, and probably with a greater sample size a definite statistical significance would have been reached. This is due to the fact that SARS-CoV-2 has affected people of varying ages, including the youngest (24). PE, on the other hand, is generally typical of an elderly population (12, 25, 26). In previous reports, an older age was associated with a longer diagnostic delay for PE in non-COVID-19 patients (19, 27).

Interestingly, there was a symptom of PE that we observed only in non-COVID-19 patients, that was the presence of palpitations. It is conceivable that the SARS-CoV-2 bradycardising action played a role in this difference (28).

Among blood tests, LDH levels tended to be more frequently altered in COVID-19 patients than in non-COVID-19 patients (89.6 and 60.8%, respectively, *p* = 0.02441). As an indicator, among other things, of severe infection, it is justifiable that LDH presented high levels in the course of SARS-CoV-2 infection. Literature reports increased LDH levels as a common evidence in COVID-19 (29, 30).

The negative impact of high LDH levels in the physician's perception of the risk of PE it is comprehensible, since it led the physician to focus on the infectious side of the disease and to interpret any other symptoms of pulmonary embolism as related to a particularly severe picture of COVID-19.

LDH values, together with fever and respiratory symptoms, are the three independent variables that in the multivariate analysis were found to be fundamental in prolonging the diagnostic delay. All three of these features, especially when present simultaneously, can be indicative of respiratory infection (29, 31, 32), and have therefore been misunderstood in SARS-CoV-2 related pneumonia. In fact, it is not surprising that a worsening in COVID-19 pneumonia was the most common misdiagnosis in our cohort.

Conversely, heart diseases were improperly diagnosed in a small number of patients, while in other studies were a frequent confounding factor for the diagnosis of PE (19).

The differences in the expression of PE between the two populations could be due to a different pathogenesis of the thrombotic event. There is evidence in the literature of local vasculitic damage at the basis of thrombotic phenomena during SARS-CoV-2 infection (33, 34). Although more than a half of the COVID-19 patients had DVT, it is possible that vasculitic damage represented, if not the cause, at least a contributing factor in the development of pulmonary embolism, generating different clinical characteristics.

We acknowledge the many limitations of this study. First, this is a single center study, conducted in a tertiary hospital in Northern Italy, thus only COVID-19 patients with a severe clinical pattern that required hospitalization have come to our attention. The delay in asymptomatic patients or patients with mild symptoms that were treated in other settings, or other geographical and climatic areas remains undefined. Also, the diagnostic approach in the emergency department and in the in-patient departments follows guidelines common to the whole hospital, so it is possible that different results have been achieved in other medical centers. A relevant feature in the evaluation of the COVID-19 patients in our hospital was to use mainly chest radiography and ultrasound as instrumental examinations. CT was not used in the first instance because of management and infectious problems. A wider use of this imaging technique would probably have reduced the delay. Second, the sample size of the study is limited, compared to the wide prevalence of both PE and COVID-19. It would certainly be interesting to integrate the data with those of other centers in order to have a greater statistical significance. Third, since COVID-19 is a new pathological condition, there is an understandable lack of knowledge that can contribute to a medical error. This is obviously a common limitation among studies about COVID-19, but it is also the reason that makes them critically important in the path toward the optimal SARS-CoV-2 management. Indeed, our data should be cautiously interpreted in the light of this specific setting, which is that of hospitalized patients with COVID-19, representing a high pre-test probability of having PE. More studies are needed for assessing generalizability of our results.

In conclusion, although its exploratory nature, this is the first study that analyzes the diagnostic delay of PE in patients affected by COVID-19, its confounding factors and potential effects on outcome. While during the past years in our center the delay was almost totally patient-related, during pandemic some COVID-19 features (mainly fever, worsening dyspnea, and persistent increased D-dimer levels) have turned away physicians from the right differential diagnosis. Another error to highlight is the scarce use of chest CT in the imaging diagnostic protocol of COVID-19 pneumonia at the time of hospital admission in our center.

Persistence of high D-dimer values over the time, contrary to what is known by literature, could be maybe a spy of a thromboembolic condition. This could be related to the different genesis of thrombosis. Indeed, in COVID-19, pulmonary inflammation induces endotheliitis and hyperactivation of coagulation cascade, causing likely local over time protracted thrombogenesis (35, 36), and rise of D-dimer values (37, 38), compatible with pulmonary thrombosis extension and persistent inflammation. However, more data are needed to define whether D-dimer value could be related to both early diagnosis and worst prognosis.

## Data Availability Statement

The original contributions presented in the study are included in the article/supplementary material, further inquiries can be directed to the corresponding authors.

## Ethics Statement

The studies involving human participants were reviewed and approved by IRCCS Policlinico San Matteo Foundation. The patients/participants provided their written informed consent to participate in this study.

## Author Contributions

ADS, FM, and MR designed and coordinated the study, interpreted data, and wrote the manuscript. SQ performed the statistical analysis. All the other authors followed up patients, locally collected data, and reviewed the paper for final approval. ADS, FM, and MVL reviewed the paper and made final critical revisions for important intellectual contents. FM, MR, SQ, FF, MVL, and ADS significantly participated in the drafting of the manuscript or critical revision of the manuscript for important intellectual content and provided approval of the final submitted version.

## Funding

This article received funding from Italian Ministry of Health, Rete Aging to MVL and ADS.

## Conflict of Interest

The authors declare that the research was conducted in the absence of any commercial or financial relationships that could be construed as a potential conflict of interest.
