# Corrigendum: Diagnostic Delay of Pulmonary Embolism in COVID-19 Patients

**DOI:** 10.3389/fmed.2022.884680

**Published:** 2022-03-23

**Authors:** Federica Melazzini, Margherita Reduzzi, Silvana Quaglini, Federica Fumoso, Marco Vincenzo Lenti, Antonio Di Sabatino

**Affiliations:** ^1^Department of Internal Medicine, San Matteo Hospital Foundation, University of Pavia, Pavia, Italy; ^2^Department of Electrical, Computer, and Biomedical Engineering, University of Pavia, Pavia, Italy

**Keywords:** pulmonary embolism, SARS-CoV-2, diagnostic delay, thrombosis, misdiagnosis

In the original article, we neglected to include in **Funding** “This article received funding from Italian Ministry of Health, Rete Aging to MVL and ADS.”

The authors apologize for this error and state that this does not change the scientific conclusions of the article in any way. The original article has been updated.

## Publisher's Note

All claims expressed in this article are solely those of the authors and do not necessarily represent those of their affiliated organizations, or those of the publisher, the editors and the reviewers. Any product that may be evaluated in this article, or claim that may be made by its manufacturer, is not guaranteed or endorsed by the publisher.

